# Editorial: Neural and behavioral mechanisms of social learning

**DOI:** 10.3389/fnhum.2025.1566408

**Published:** 2025-02-13

**Authors:** Laura A. Agee, Marie-H. Monfils, Abdellah Fourtassi

**Affiliations:** ^1^Department of Neuroscience, The University of Texas at Austin, Austin, TX, United States; ^2^Department of Psychology, The University of Texas at Austin, Austin, TX, United States; ^3^Aix Marseille University, Université de Toulon, CNRS, LIS, Marseille, France

**Keywords:** social learning, social behavior, social isolation, social threat learning, decision making, social context

Social learning, the acquisition of new information or behavior through observation of or instruction by other organisms, has been observed in a host of species (Laland, 2004; Brown and Laland, 2003; Wilkinson et al., 2010). Humans in particular rely heavily on social learning strategies to acquire and distribute information between individuals and across generations (Dean et al., 2014). Moreover, access to social learning opportunities is essential for normative behavioral and cognitive development, as is evidenced by the persistent deficits observed in individuals deprived of social contact in early life. In accordance with the clear importance of this information transfer method, much research has been dedicated to understanding social learning at a mechanistic level (Monfils and Agee, 2019; Olsson et al., 2020; Ollendick and King, 1991; Heyes, 2012). In this editorial, we feature a collection of recent articles focused on further developing our understanding of the behavioral and/or biological underpinnings of social learning.

In the Research Topic's first article, de Groot et al. assessed human participants on their reliance on social information and utilized magnetic resonance imaging (MRI) to calculate the total volumes of various brain regions. Using machine learning models, they attempted to determine whether the total volume of different brain regions related to the degree of reliance on socially acquired information. They found that increased reliance on information thought to be coming from another individual for decision making was related to higher volume in the pars triangularis and entorhinal cortex. They also found a negative correlation between reliance on social information and activity in certain regions of the frontal and post-central gyri. While the authors speculated that the postcentral and frontal gyri were more likely to be mediating visual processes required for task performance, the other regions were thought to be uniquely involved in social learning.

In their recent methods article, McTaggart et al. describe the development of an open-source automated social interaction chamber for the study of social threat learning in mice. Their device consists of a small “social stimulus” chamber—large enough to house an adult mouse—that can neatly slot into standard modular fear conditioning chambers. A series of infrared photobeams at the barrier between the two chambers detect interactions between a stimulus and test mouse, allowing for shock delivery to the test mouse timed to social interaction. They demonstrate that this system successfully induces learned social avoidance in mice shocked on interaction with the stimulus mouse. Their design allows for easy integration of social threat learning as a behavioral model into any lab outfitted with modular fear conditioning chambers. McTaggart et al.'s exciting research paves the way for labs focused on observational social fear to study how learned social avoidance effects observational fear learning in mouse models.

Villalon et al. present novel research focused on the effects of social environment, specifically social isolation, on asocial learning. This study extends recent research from the lab demonstrating impaired learned avoidance of noxious thermal stimuli in socially stressed male mice (Felix-Ortiz et al., 2024). In this new study, the same stressor and paradigm are extended to both male and female mice. The impairment in thermal safety learning was found to extend to female mice as well, though the degree of impairment was similar between the sexes.

Finally, an opinion piece by Agee et al. highlights the potential confounding effects of failing to properly control for social context effects in experimental studies. We briefly review the human and animal literature, examining how the different aspects of social environment that test subjects are exposed to effects behavior. Included in this discussion are the potential for social learning via emotional contagion or experimenter presence/behavior (particularly in human research) to bias subject responding. We conclude with an overview of strategies for avoiding such confounds in future research.

Much about the behavioral and biological underpinnings of social learning remain to be understood. The articles included in this Research Topic represent some of the latest findings, methodological advances, and discussions that may help further elucidate this Research Topic.
